# Facial Onset Sensory and Motor Neuronopathy: New Cases, Cognitive Changes, and Pathophysiology

**DOI:** 10.1212/CPJ.0000000000000834

**Published:** 2021-04

**Authors:** Eva M.J. de Boer, Andrew W. Barritt, Marwa Elamin, Stuart J. Anderson, Rebecca Broad, Angus Nisbet, H. Stephan Goedee, Juan F. Vázquez Costa, Johannes Prudlo, Christian A. Vedeler, Julio Pardo Fernandez, Mónica Povedano Panades, Maria A. Albertí Aguilo, Eleonora Dalla Bella, Giuseppe Lauria, Wladimir B.V.R. Pinto, Paulo V.S. de Souza, Acary S.B. Oliveira, Camilo Toro, Joost van Iersel, Malu Parson, Oliver Harschnitz, Leonard H. van den Berg, Jan H. Veldink, Ammar Al-Chalabi, Peter N. Leigh, Michael A. van Es

**Affiliations:** Universitair Medisch Centrum Utrecht (EMJB, HSG, JI, MP, LHB, JHV, MAE), Department of Neurology, Utrecht, The Netherlands; Brighton and Sussex Medical School (AWB, ME, RB, PNL), Clinical Imaging Sciences Centre, Brighton, United Kingdom; Hurstwood Park Neurological Centre (AWB, ME, SJA, RB, AN), Haywards Heath, United Kingdom; Hospital Universitari i Politècnic La Fe (JFVC), ALS Unit, Department of Neurology, Valencia, Spain; Centro de Investigación Biomédica en Red de Enfermedades Raras (CIBERER) (JFVC), Madrid, Spain; Department of Neurology (JP), Rostock University Medical Center and German Center for Neurodegenerative Diseases (DZNE), Germany; Department of Neurology (CAV), Haukeland University Hospital and Department of Clinical Medicine, Bergen, Norway; Department of Neurology (JPF), Hospital Clínico Universitario de Santiago, Santiago, Spain; Department of Neurology (MPP, MAAA), Hospital Universitari de Bellvitge, Barcelona, Spain; ALS/MND Centre (EDB, GL), 3rd Neurology Unit, Fondazione IRCCS Institute Neurologico Carlo Besta, Milan, Italy; Department of Biomedical and Clinical Sciences “Luigi Sacco” (GL), University of Milan, Milan, Italy; Department of Neurology and Neurosurgery (WBVRP, PVSS, ASBO), Federal University of São Paulo (UNIFESP), São Paulo, Brazil; National Institutes of Health (CT), National Human Genome Research Institute, Bethesda, United States of America; Memorial Sloan Kettering Cancer Center (OH), NY; King's College Hospital NHS Foundation Trust (AA-C), London, United Kingdom; and Department of Neuroscience (PNL), Brighton and Sussex Medical School, Brighton, United Kingdom.

## Abstract

**Purpose of Review:**

To improve our clinical understanding of facial onset sensory and motor neuronopathy (FOSMN).

**Recent Findings:**

We identified 29 new cases and 71 literature cases, resulting in a cohort of 100 patients with FOSMN. During follow-up, cognitive and behavioral changes became apparent in 8 patients, suggesting that changes within the spectrum of frontotemporal dementia (FTD) are a part of the natural history of FOSMN. Another new finding was chorea, seen in 6 cases. Despite reports of autoantibodies, there is no consistent evidence to suggest an autoimmune pathogenesis. Four of 6 autopsies had TAR DNA-binding protein (TDP) 43 pathology. Seven cases had genetic mutations associated with neurodegenerative diseases.

**Summary:**

FOSMN is a rare disease with a highly characteristic onset and pattern of disease progression involving initial sensory disturbances, followed by bulbar weakness with a cranial to caudal spread of pathology. Although not conclusive, the balance of evidence suggests that FOSMN is most likely to be a TDP-43 proteinopathy within the amyotrophic lateral sclerosis–FTD spectrum.

Facial onset sensory and motor neuronopathy (FOSMN) is a rare neurologic syndrome first described by Vucic et al. in 2006.^1^ It has a characteristic phenotype with paresthesia and numbness arising within the trigeminal nerve distribution, which slowly spreads to the scalp and thereafter descends to the neck, upper trunk, upper extremities, and in some cases to the lower extremities. The initial sensory disturbance may begin in a perioral location, or affect one or more of the trigeminal branches unilaterally and then become bilateral, or be bilateral from the beginning. Lower motor neuron features present later or concurrently with the sensory deficits. These include dysphagia, dysarthria, fasciculations, muscle weakness, and muscle atrophy, along the same rostral-caudal direction.^2^ In general, only the lower motor neuron system is involved, although upper motor neuron signs have been reported in a few cases.^3–11^

Pathogenesis is uncertain. Initial indications suggested an autoimmune basis since autoantibodies have been reported as well as a partial and subjective response to immunotherapy.^1,3,4,6,8,12–16^

The finding of TAR DNA-binding protein (TDP) 43 pathology at autopsy in several FOSMN cases, however, implies a neurodegenerative mechanism and a likely association with amyotrophic lateral sclerosis (ALS).^8–10,17^

The objective of this study is to expand our understanding of FOSMN by evaluating clinical findings and the natural history of the disease in a larger sample than hitherto available and to review the current literature.

## Methods

### Updated Case Series

FOSMN is an extremely rare disorder, with only 38 cases reviewed to date.^2^ We provide an updated case series by describing new incident cases and cases identified through literature search.

Incident cases were seen at the following specialized (neuromuscular) clinics: University Medical Centre Utrecht (The Netherlands), Brighton, and Sussex University Hospitals NHS Trust (UK), Rostock University Hospital (Germany), University Hospital i Politècnic La Fe Valencia (Spain), University Hospital Clínico de Santiago (Spain), University Hospital de Bellvitge in Barcelona (Spain), University Hospital Haukeland Bergen (Norway), Foundation of the Carlo Besta Neurological Institute IRCCS Milan (Italy), National Human Genome Research Institute, Bethesda (United States of America), and the Federal University of São Paulo (Brazil).

Several patients self-reported to us, some through the FOSMN patient organization, in which case the treating physician was contacted to obtain medical records. At present, there are no formal diagnostic criteria for FOSMN. The diagnosis was therefore made based on excluding other potential causes and expert opinion.

**Figure 1:**
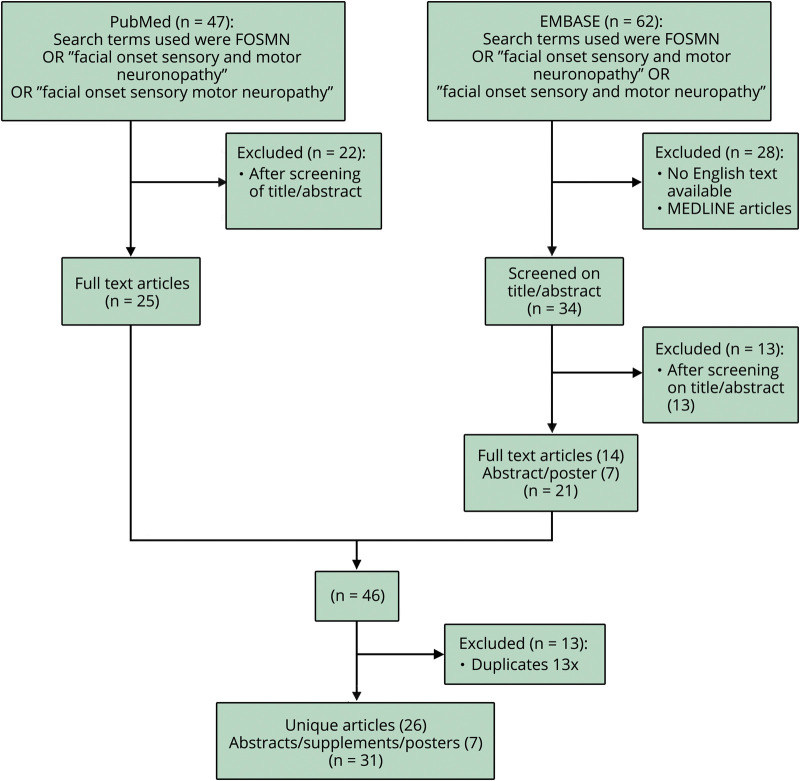
Summary of the Results from the Literature Search The search was performed on September 1, 2019, using PubMed and EMBASE on articles from January 1, 2006, to September 1, 2019, reporting on FOSMN. Titles and abstracts were screened, and relevant full-text articles were retrieved. References were screened for additional studies. FOSMN = facial onset sensory and motor neuronopathy.

We performed a systematic search of the literature with the objective of identifying FOSMN cases that were not included in previous reviews. Titles and abstracts were screened, and relevant full-text articles were retrieved. Corresponding authors were also contacted for additional details (figure 1). Published cases, not included in reviews before, and our incident cases will be reported separately as novel cases.

### Clinical Data

We extracted the following data from published cases and the medical records of the incident cases: sex, age at onset, disease duration, bulbar signs, sensory deficits, weakness of extremities, upper motor neuron signs, laterality, cerebral spinal fluid results, antibody testing, results of imaging, genetic analysis, neurophysiologic evaluation, type of therapy and response, biopsy, need for a gastric feeding tube or percutaneous endoscopic gastrostomy, need for noninvasive ventilation, cause of death, and autopsy findings.

Follow-up data on previously published cases and available longitudinal data from the incident cases were included.^13,16,18^ We encoded and stored the data in a secure, password-protected database.

### Pathophysiology

There are 2 leading hypotheses with regard to the cause of FOSMN, namely, that it is either a neurodegenerative or an autoimmune disorder. From our cases and the literature, we extracted data on ancillary investigations, response to treatment, disease course, and postmortem findings. These data were subsequently categorized as supportive of a neurodegenerative or autoimmune etiology.

## Results

### Updated Case Series and Clinical Findings

**Figure 2:**
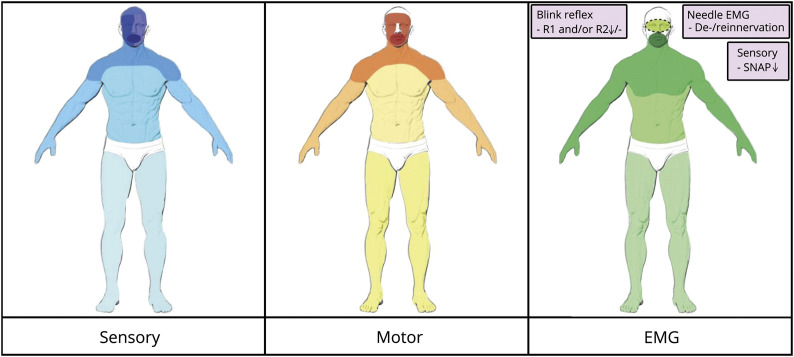
Summary Distribution Abnormalities in FOSMN Distribution of sensory, motor, and electrophysiologic findings in patients with FOSMN. In all 3 pictures, the darker color represents the higher amount of patients with abnormalities, showing the cranial-caudal spreading. EMG = electromyography; FOSMN = facial onset sensory and motor neuronopathy; SNAP = sensory nerve action potential.

We identified a total of 29 incident cases. Our literature search yielded 26 full-text articles reporting on a total of 64 patients with FOSMN, of which 29 were not described in a review before.^1,3–16,18–24^ We further identified 7 abstracts/posters/supplements reporting on 11 potential cases and 2 articles mentioning 10 FOSMN cases without further patient characteristics.^25,26^ After reaching out to the corresponding authors, we received a detailed poster on 7 patients with FOSMN.^17^ We chose not to include the other 14 possible patients with FOSMN due to the limited information in the abstracts. This led to a total of 100 patients with FOSMN. The main clinical findings of the 28 cases that had not been reported previously are summarized in table e-1, links.lww.com/CPJ/A171. The baseline characteristics for all 100 FOSMN cases are described in table 1. Most cases developed progressive sensory deficits, starting in the trigeminal nerve distribution and slowly spreading to the scalp, neck, shoulders, upper extremities, and in a few cases to the lower extremities. Later (in some cases concurrently), lower motor signs developed, starting in the face and spreading through the same pattern as the sensory disturbances, resulting in bulbar symptoms (dysarthria and dysphagia), fasciculations, atrophy, and weakness of the involved muscles (figure 2). Asymmetrical presentation of the symptoms (initially) occurred in 48 of the cases, in whom the first affected side remained more severely affected as the disease progressed despite involvement of the contralateral side. Lower motor neuron signs were observed in all FOSMN cases. Upper motor neuron signs, such as brisk reflexes, Babinski sign, and clonus, were reported in 27 cases.^3–11,14^

**Table 1:**
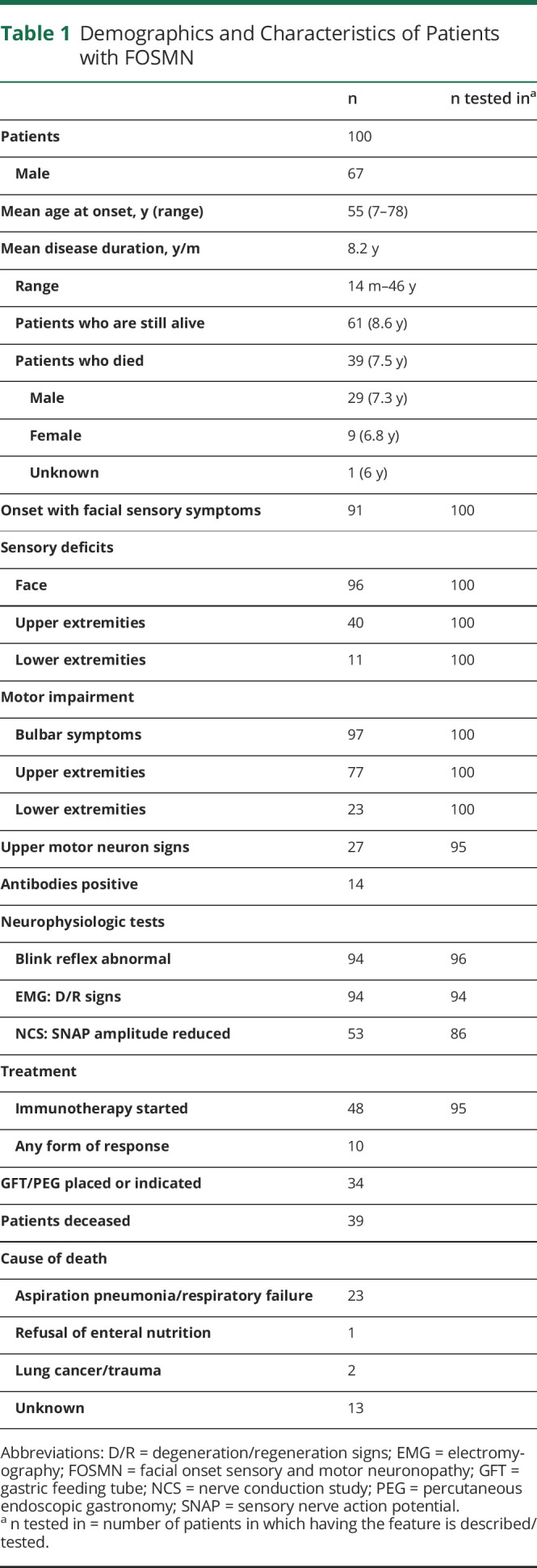
Demographics and Characteristics of Patients with FOSMN

### Natural history

The mean age at onset is approximately 55 years (range 7–78 years). The rate of progression is highly variable, and survival ranges from 14 months to over 46 years.^1,3–23^ Ninety-one percent of the cases started with progressive facial sensory impairment, followed by motor involvement. In a single case, sensory symptoms slowly developed 10 years after onset of the motor deficits.^13^ Two patients developed high-frequency sensorineural hearing loss developed, which started mid-50s (cases 1 and 2).^13^ Case 4 from our case series (table e-1, links.lww.com/CPJ/A171) also complained of hearing loss; however, results of ancillary investigations were not available. Sleeping problems were described in 2 cases; case 9 had signs suggesting REM sleep behavioral disorder, and in case 1,^13^ this was confirmed by polysomnography. (Pseudo)choreoathetosis, was seen in 6 patients (cases 21, 22, 25, 26, 27, and a literature patient who was followed up on).^16^

Ninety-seven percent of the patients developed bulbar symptoms.^1,3–11,13,14,16–23^ Weight loss resulting from dysphagia in FOSMN may be severe, and in 34 cases, gastrostomy was indicated.^1,3,4,7,8,10,11,13,14,16–19,23^ The mean disease duration before placement was 5 (1–14) years after onset of the symptoms. Fourteen patients required noninvasive ventilation.^8,11,16,19^

Of the 39 patients who died, the mean disease duration was 7.5 years. Of the known causes of death, 23 patients died as a result of progressive bulbar weakness, leading to aspiration pneumonia or respiratory failure.^3,5,7,8,10,12,17,20,21,23^

### Behavioral Changes and Cognitive Impairment

Five patients developed progressive behavioral changes and were clinically diagnosed with comorbid behavioral variant frontotemporal dementia (bvFTD) by the treating neurologist. Review of the medical records indeed shows that the Rascovsky criteria are met.^27^ One patient also showed progressive behavioral changes strongly suggestive of bvFTD, but no formal diagnosis was made. Considering FOSMN is a potential mimic (or perhaps even an atypical formal form) of ALS, patients are commonly referred to motor neuron disease (MND) clinics. Given that ALS and FTD are highly related disorders, screening for cognitive and behavioral changes has become part of the standard workup in MND, most frequently using the Edinburgh Cognitive and Behavioral ALS screen (ECAS). Therefore, ECAS data for 6 FOSMN cases, seen at MND clinics, were available to us (one of the patients diagnosed with bvFTD also underwent an ECAS). In total, neuropsychological data were available for 11 patients, of which 8 demonstrated changes within the FTD spectrum (72%). Three patients showed normal ECAS results and no behavioral changes.

There are no available data on cognition and behavioral changes in the remaining 89 cases; therefore, we are unable to provide an estimate of the frequency of these changes in FOSMN. A brief summary of neuropsychological findings of these cases is provided in table 2.

**Table 2:**
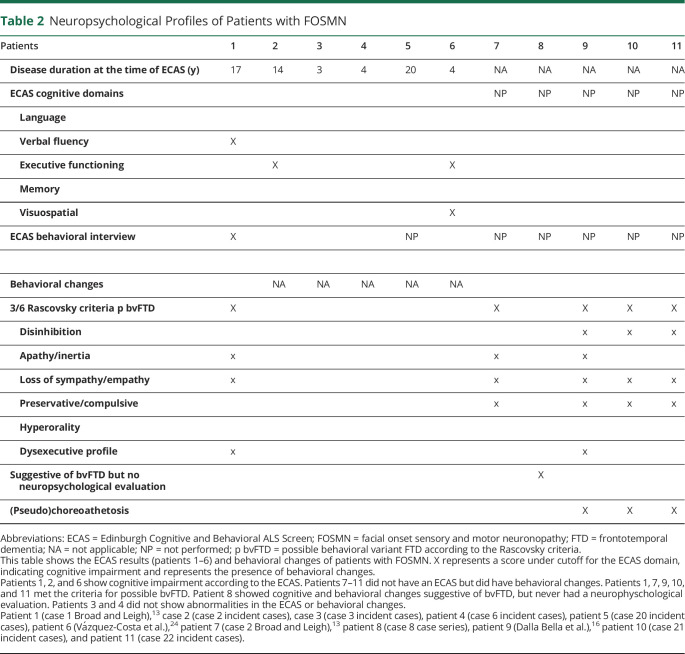
Neuropsychological Profiles of Patients with FOSMN

The first case is a man (patient 1),^13^ who at age 59 years (17 years after onset) began to complain of deteriorating memory for recent events, had difficulty planning activities, and became apathetic with blunted empathy or emotional responses, thereby meeting the criteria for possible bvFTD. Cognitive screening using the ECAS did not demonstrate memory deficits, but did show that he scored under the cutoff for verbal fluency.

The second case is a 45-year-old man (patient 7),^13^ who 12 years after onset of FOSMN became very irritable with social withdrawal and became obsessive and mildly apathetic. Memory for recent events and concentration abilities were also diminished. He also meets the criteria for possible bvFTD.

Another case with signs of bvFTD is patient 8. At age 69 years, after 22 years of disease, he developed short-term memory loss, aphasia, and difficulty following instructions. Episodes of confused nocturnal wandering occurred soon after sleep onset, during which he held the delusion that his wife was an imposter and could become aggressive when challenged. Unfortunately, limited information was available from medical records, but aphasia and behavioral changes were the most prominent features in this case. It is unclear whether we should classify this patient as having primary progressive aphasia or as cognitive and behavioral impairment according to the current Strong criteria for frontotemporal spectrum disorder in MND.^28^ The latter is a term, which is applicable to patients with cognitive and behavioral changes within the spectrum of FTD, but that do not meet the formal criteria. Unfortunately, the patient died before full neuropsychological evaluation could be achieved. Brain MRIs were normal in all 3 cases.

Two patients showed cognitive impairment according to the ECAS without behavioral changes. Patient 2 and patient 6, a Spanish patient, with brain atrophy, predominating in the right frontotemporal lobes. His ECAS showed mild cognitive impairment (executive functioning), and another memory screening test (M@T) showed mild impairment in long-term episodic memory. Because the ALS-FTD-Q is not validated in Spanish, a semistructured ECAS interview and the FRSBE questionnaire were performed with normal results.^24,29^

### Differential Diagnosis

At present, there is no diagnostic test that can definitively confirm FOSMN nor are there existing diagnostic criteria. The diagnosis is based on expert opinion and relies predominantly on a history of facial onset sensory symptoms, followed by facial weakness and a subsequent cranial-caudal spread of symptoms.

Other causes need to be ruled out, making FOSMN a diagnosis by exclusion. There is considerable heterogeneity with regard to the presentation. For instance, some patients did not seek medical attention for the facial sensory signs. The interval between the onset of facial sensory signs and weakness can range from years to simultaneous onset. There was 1 case where subtle sensory symptoms only developed 10 years after onset of the motor deficits.^13^ The rate of progression differs considerably, with some patients developing severe bulbar weakness within a year compared with patients with exclusively sensory symptoms for a decade. Therefore, disease duration, rate of progression, and chief complaint (sensory, motor, or both) influence the differential diagnosis and the workup.

In patients with isolated sensory signs, idiopathic trigeminal neuropathy or a central lesion is the main alternative. In cases in which bulbar weakness is progressive or the predominant feature, ALS (and ALS mimics) needs to be considered, particularly because it has been suggested that FOSMN is an atypical form of ALS.^30^ In most cases, the differential diagnosis is quite extensive. Table 3 provides an overview of the differential diagnosis and most relevant additional investigations.

**Table 3:**
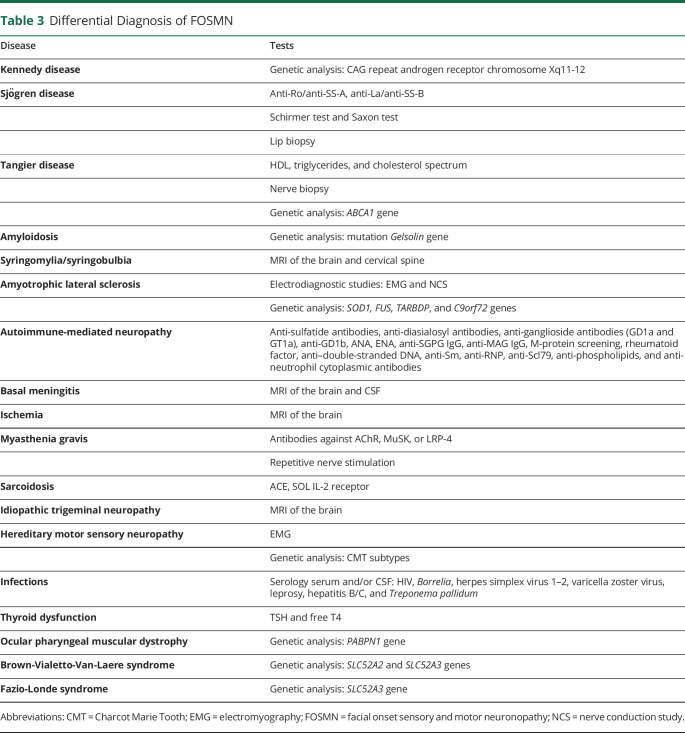
Differential Diagnosis of FOSMN

Laboratory investigations are generally unremarkable. Mildly elevated CK levels may be found, but never exceeding >5 × upper limit normal. CSF is also normal. MRI studies in patients with FOSMN do not explain the symptoms, although mild to moderate midcervical cord atrophy has been reported in several cases, bright tongue sign in 3 patients,^11^ and frontotemporal atrophy in 2 patients.^24^

Extensive genetic testing has been performed in various cases, which included ALS genes (*C9orf72, FUS, SOD1, TARDBP*), oculopharyngeal muscular dystrophy (*PABPN1*), Kennedy disease (*AR*), panels for spinocerebellar ataxia as well as whole-exome sequencing. With a few exceptions (discussed below), genetic testing has not given any insight so far.

There are several electrophysiologic findings that support a diagnosis of FOSMN. Blink reflexes are abnormal in all cases but 2 cases,^17^ varying from unilateral to bilateral delayed or absent R1 and/or R2 responses. Needle electromyography showed signs of denervation and/or reinnervation in all patients, spreading in a cranial-caudal distribution. Sensory nerve conduction studies show reduced sensory nerve action potential amplitudes of the upper limbs in 62% and in the lower limbs in 4 cases (figure 2).

### Pathophysiology

The main hypotheses are that FOSMN is either a neurodegenerative or an autoimmune disorder. We reviewed the literature and extracted data on ancillary investigations, response to immune-modulating treatment, genetics, disease course, and postmortem findings and subsequently categorized these data as supportive of a neurodegenerative or autoimmune etiology (table 4). Autoantibodies were present in 14 patients. Three of these cases, 1 with low-positive ANA and anti-Ro, 1 with anti-sulfo-glucuronyl paraglo-boside IgG, anti-myelin-associated glycoprotein IgG, and the final patient with a monoclonal gammopathy, responded to immunotherapy.^6,8,14^ One patient had apparent stabilization of symptoms,^6^ 1 had partial response of weakness and sensory impairment but thereafter had worsening respiratory symptoms,^8^ and in the last patient, intravenous immunoglobulin relieved her facial numbness and improved the blink reflex, but motor symptoms worsened. Six other patients also had a form of response to immunotherapy, but did not report positive immunologic studies.

**Table 4:**
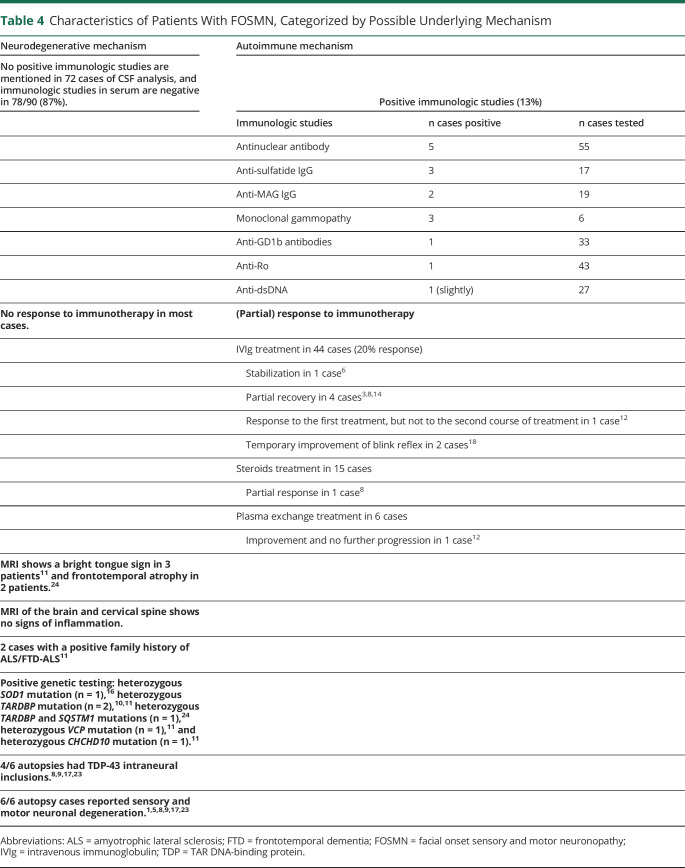
Characteristics of Patients With FOSMN, Categorized by Possible Underlying Mechanism

In a total of 6 patients with FOSMN, an autopsy was performed.^1,5,8,9,17,23^ The pathologic studies show loss of motor neurons in the facial nerve nucleus and hypoglossal nucleus and cervical anterior horns and loss of sensory neurons in the main trigeminal sensory nucleus, nucleus of the solitary tract, and dorsal root ganglia. Four cases had TDP-43–positive glial inclusions.^8,9,17,23^ The other 2 cases had no intraneural inclusions and stained negative for ubiquitin.^1,5^ Cutaneous nerve and muscle biopsies show a loss of myelinated fibers and Wallerian degeneration of the nerves without signs of vasculitis or amyloid deposition.^1,4,5,17,20,21^ Genetic analysis was positive in 7 cases. In 1 case, a heterozygous D90A *SOD1* mutation (familial ALS) was found. There were also 2 cases of a heterozygous *TARDBP* mutation,^10,11^ of which 1 patient had a positive family history (2 maternal cousins with definitive genetic diagnosis of ALS and mother with FTD).^11^ There was 1 case of a heterozygous mutation in *TARDBP* and in *SQSTM1*.^24^ Further mutations found were a heterozygous *VCP* mutation in a patient with a mother with FTD, brother with spinal-onset ALS, and nephew with IBM^11^; a patient with a heterozygous *CHCHD10* mutation, whose mother had bilateral eyelid ptosis and Parkinson disease and brother had chronic exercise intolerance and proximal myopathy^11^; and 1 case with a genetic mutation in the *PABPN1* gene (oculopharyngeal muscular dystrophy).^5^ Furthermore, 2 patients with FOSMN were described who underwent whole-exome sequencing without definitive results, but had a positive family history of neurodegenerative diseases, a mother with FTD-ALS, and a maternal aunt and grandmother with Parkinson disease, and a patient with 2 brothers and a mother with ALS.^11^ A positive family history was also seen in incident case 13 (brother died of ALS) and incident case 16 (FTD). To date, no cases with a positive family history of FOSMN are reported.

## Discussion

FOSMN is a rare neurologic syndrome, with a total of 100 documented patients worldwide to date. It typically commences with a trigeminal distribution sensory disturbance, which progressively engulfs the head, neck, trunk, and upper and lower extremities. It is followed by motor weakness spreading along the same craniocaudal distribution, progressing to bulbar symptoms, weakness of the extremities, and respiratory motor impairment (figure 2). Over the course of the disease, patients with FOSMN develop dysphagia leading to severe weight loss (requiring gastronomy) as well as sometimes fatal aspiration pneumonia. Causes of death in FOSMN are similar to ALS, adding to the view that there is overlap between the 2 conditions.

Hitherto, little attention has been paid to the presence of behavioral and cognitive changes in patients with FOSMN. Six cases are described to have behavioral changes, suggestive of bvFTD. In 2 patients without behavioral changes, evidence for executive dysfunction was seen on the ECAS. It seems that behavioral changes and cognitive changes within the FTD spectrum may occur in patients with long disease duration, expanding the phenotype of FOSMN. Additional neuropsychological research in FOSMN seems warranted to better understand these cognitive and behavioral changes.

Because the cause of FOSMN is unknown, there is no test that definitively confirms this diagnosis. Patients are therefore diagnosed based on expert opinion and by excluding other disorders that could cause comparable symptoms. Because there are no formal diagnostic criteria, we cannot be sure that all documented patients indeed have FOSMN. Three patients have no sensory symptoms, and 1 patient only has sensory symptoms in the lower extremities. They do however all have abnormal blink reflexes indicating trigeminal involvement. Until consensus is reached on formal diagnostic criteria that have been validated, the diagnosis is based on expert opinion, potentially causing bias in the present findings.

Thus far, the pathogenesis of FOSMN has remained elusive. There are 2 main hypotheses. It has been suggested that the underlying mechanism may be either autoimmune mediated or neurodegenerative.

An autoimmune origin is supported by the fact that autoantibodies were present in 14 patients with FOSMN. It must be noted that the panel of antibodies that was tested varied considerably between cases and that therefore the percentage of positive cases could be an underestimation or that yet unidentified antibodies are involved. However, there seems to be little consistency in the detected antibodies, and several of these antibodies are associated with multiple conditions and may be even be found in healthy individuals. Therefore, these antibodies findings seem to be nonspecific. A potential underlying autoimmune cause could possibly be detected with better antibody screening or by using induced pluripotent stem cell–derived trigeminal neurons.^31^

Immune-modulating therapy is often initiated in FOSMN, and (partial) response has been reported, which would also support an autoimmune etiology. The definition of treatment response was rather arbitrary, ranging from partial recovery/stabilization to a temporary improvement of the blink reflex. In several cases, the treatment response was not documented by objective measurement (and therefore may have been merely subjective) or perhaps not clinically relevant (temporary improvement of blink reflex), making the effect debatable. There have been no placebo-controlled studies of immunotherapy.

Multiple lines of evidence seem to support the hypothesis that FOSMN is a neurodegenerative disorder. Perhaps most convincing is that the autopsies of 4 FOSMN cases revealed intraneuronal TDP-43 inclusions, which is the pathologic hallmark of ALS and FTD.

In a journal supplement, 2 patients with FOSMN are mentioned, in which postmortem examination also revealed TDP-43 aggregates in the motor and sensory trigeminal nucleus and anterior horn cells of the cervical and thoracic cord in patient 1 and in the sensory and motor trigeminal nucleus and cervical, thoracic, and lumbar motor neurons, with associated astrocytosis, in patient 2.^32^ Unfortunately, we only had access to the abstract, but these findings also support the neurodegenerative etiology.

Furthermore, a link to ALS has been suggested after a patient with FOSMN was found to harbor a heterozygous mutation in the familial ALS gene for *SOD1*. This specific mutation (D90A) generally causes a recessive form of ALS, although there are several pedigrees in which this mutation appears to cause disease in an autosomal dominant manner. It is therefore unclear whether this mutation should be considered pathogenic.^33^ Other cases of FOSMN with mutations related to ALS have also been found. Two cases with a heterozygous *TARDBP* mutation, both considered pathogenic,^10,11^ a heterozygous pathogenic *VCP* mutation and a heterozygous pathogenic *CHCHD10* mutation.^11^ Finally, there was a patient with 2 heterozygous mutations in the *TARDBP* and in *SQSTM1* genes. In this case, it was suggested that the combination of the mutations resulted in this phenotype.^24^ Some of these patients had a positive family history of ALS or other neurodegenerative diseases. Four patients with a positive family history of FTD-ALS, ALS, FTD, and or Parkinson disease, but without definitive results from genetic testing, were also reported.^11^ This suggests that these diseases may cluster with pedigrees, like other neurodegenerative diseases.^34,35^

The fact that patients develop severe bulbar symptoms with respiratory insufficiency (reminiscent of ALS) and may develop cognitive and behavioral changes within the FTD spectrum supports considering FOSMN as part of the FTD-MND continuum. Indeed, findings at autopsy have also provided evidence that FOSMN may be a TDP-43 proteinopathy.

TDP-43 pathology is seen in approximately 98% of patients with ALS, but is absent in the 2% of ALS that can be attributed to *SOD1* mutations.^33,36^ The presence of TDP-43 pathology and the identification of an *SOD1* mutation suggest a connection to ALS, and although there is some limited evidence in support of an autoimmune mechanism, this is far from conclusive. Our tentative conclusion is that FOSMN is most likely to have a neurodegenerative etiology, possibly within the ALS-FTD continuum.

To determine the underlying cause of FOSMN, more research is needed through an international collaboration considering the rare nature of the disease. The development of diagnostic criteria followed by validation is an important first step. A better understanding of the natural history of the disease will aid physicians in providing their patients with the appropriate care. Further characterization of the disease, in particular whether FOSMN is a TDP-43 proteinopathy, may provide inroads into therapy options based on insights from other TDP-43–related disorders.

**TAKE-HOME POINTS**

→ FOSMN is an extremely rare neurologic disease starting with sensory impairment in the face spreading along a craniocaudal distribution, followed by motor involvement spreading by the same pattern.
→ FOSMN resembles ALS in the sense that motor impairment results in weakness, eventual need for gastronomy, and in most cases respiratory failure. Disease duration, however, is longer than for patients with ALS.
→ Although not conclusive, the balance of evidence suggests that FOSMN is most likely to be a TDP-43 proteinopathy within the ALS-FTD spectrum. This is supported by the resembling disease progression, presence of TDP-43 pathology, and genetic mutations associated with MNDs found in patients with FOSMN.
→ Further (international) research is required to form diagnostic criteria and to obtain further knowledge on FOSMN.
